# Epigenetic manipulation of anterior insular cortex alters neural signals and cognitive control

**DOI:** 10.1038/s41386-025-02181-5

**Published:** 2025-08-03

**Authors:** Sarah Perry, Paul S. Sharalla, Dylan R. Sarubin, Xuan Li, Matthew R. Roesch, Adam T. Brockett

**Affiliations:** 1 Department of Psychology, University of Maryland, College Park, MD USA; 2 Program in Neuroscience and Cognitive Science, College Park, MD USA; 3 Department of Biological Sciences, University of New Hampshire, Durham, NH USA

**Keywords:** Epigenetics and behaviour, Cognitive control, Neuronal physiology

## Abstract

The balance between impulsive prepotent behavior and inhibition is a crucial aspect of self-control, and disruptions to this balance are observed in aging and various neuropsychiatric conditions, such as addiction. Both the insula and histone deacetylases (HDACs), a family of epigenetic enzymes, are implicated in these disruptions, with HDAC inhibitors showing therapeutic potential. However, the role of single neuron activity in the insula in relation to cognitive control and how this activity is affected by HDAC modulation in behaving animals remains unclear. In this study, we focus on HDAC5, a class II HDAC that regulates gene transcription and shuttles between the nucleus and cytoplasm in response to neuronal activity. We investigate how overexpression of nuclear-localized HDAC5 in the anterior insula influences cognitive control and associated neural processes in rats performing a STOP-change task. This task contrasts frequent prepotent responding (GO trials) with infrequent response inhibition and behavioral redirection (STOP-change trials). Our results show that HDAC5 overexpression altered neural activity linked to executive control signals in the anterior insula, leading to faster prepotent responses and a reduced capacity for behavioral inhibition (i.e., increased motor impulsivity). Additionally, while insula firing was influenced by trial history (i.e., previous trial rewarded or not), HDAC5 overexpression did not affect this feature. These findings suggest that increased HDAC5 activity impair cognitive control, and targeting HDAC5 in this region may offer a promising therapeutic approach for enhancing executive control and mitigating impulsivity-related cognitive, emotional, and social impairments.

## Introduction

Dysregulated gene transcription is observed across the lifespan, and many neuropsychiatric disorders may be attributed to aberrant epigenetic enzyme activity [1–8]. Among these epigenetic enzymes are histone deacetylases (HDACs), which deacetylate histones to increase electrostatic affinity between histones and DNA, resulting in reduced gene transcription rates [9–11]. While some negative regulation is needed for appropriate learning and memory functions, too much or too little may negatively disrupt normal cognitive processes. Gene-environment interactions add a layer of complexity when considering epigenetic mechanisms, as life experiences and age can alter epigenome signaling [12–24].

Here, we focus on HDAC5, a class II HDAC that shuttles between the nucleus and cytoplasm in response to intracellular signaling and is highly expressed in cortical regions [25–27]. Inhibition of class II HDACs has been shown to increase brain-derived neurotrophic factor expression in vitro [28, 29] and improve mood and cognitive impairments [30–33], demonstrating an interesting relationship between HDACs, neural plasticity, and more generally, brain functions. Although there is increasing research looking at the role of HDACs in drug addiction [10, 34–40] and memory [24, 27, 41], few studies have explored how HDAC manipulation impacts executive control and related neural firing.

To address this gap, we recorded from anterior insula as rats performed a STOP-change task. This task builds an automatic prepotent tendency to respond quickly on GO trials, which account for the majority of trials (80%), and then, on a smaller subset of trials (20%), inhibit and redirect behavior in the opposite direction. Automaticity on GO trials and the ability to inhibit behavior on STOP trials has been shown to be altered during aging and numerous psychiatric illnesses, and work in humans has implicated insula in functions necessary for STOP-signal performance [42–45]. However, to our knowledge, no one has recorded from insula in rats performing a STOP-change task or determined how firing is altered by epigenetic manipulation.

Here, we report on neural correlates related to cognitive control in the insula and how they are altered in rats with overexpression of a nuclear-localized HDAC5. We show that rats with HDAC5 overexpression were faster on GO trials and worse on STOP trials (i.e., increased impulsivity). Consistent with these behavioral changes, neural directional behavioral response signals were increased and decreased in HDAC5 rats. These results suggest that increased HDAC5, known to reduce gene transcription, can increase motor impulsivity via changes in executive control signals in the insula.

## Materials and methods

### Animals

Sprague-Dawley rats (250–275 g) rats obtained from Charles River were held and tested at University of Maryland according to university and NIH guidelines. Rats were housed on a 12-h light-dark schedule. Experiments were approved by IACUC and conformed to the National Research Council Guide of the Care and Use of Laboratory Animals.

### Procedures and histology

**Fig. 1:**
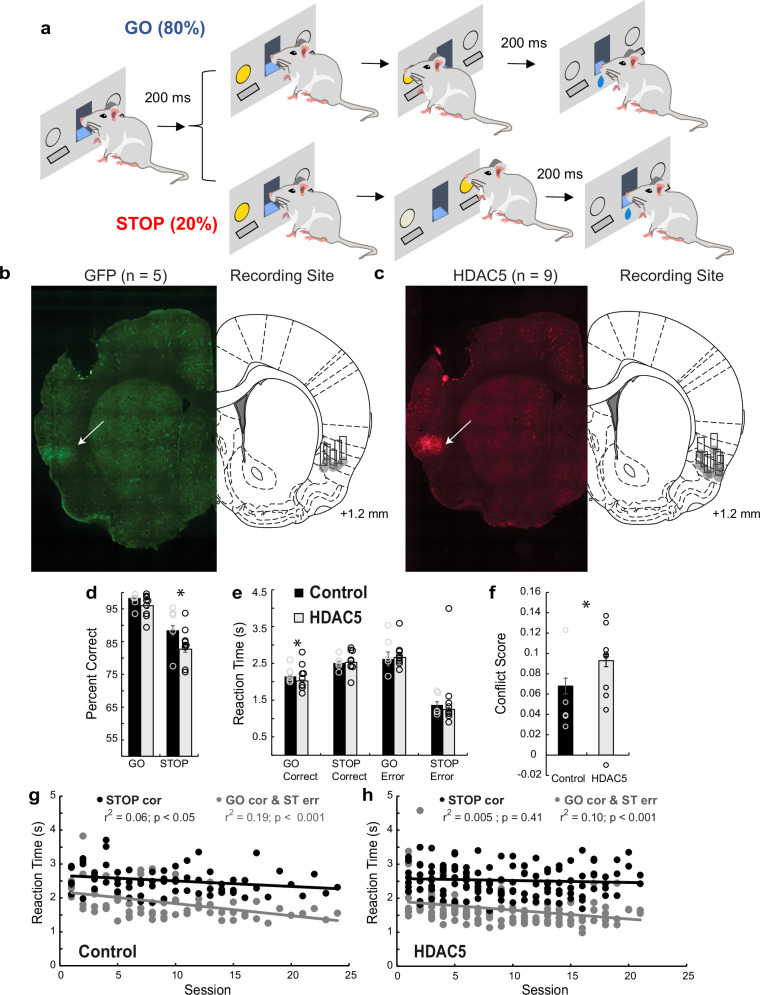
HDAC5 in the insula disrupts accuracy and increases speed on GO trials. **a** Overview of the general task sequence. Following house light illumination, rats held a nose poke in the central fluid well for 200 ms at which point a directional cue light illuminated on either the left or right side. On 80% of trials (GOs; blue), this light indicated the direction the rat can respond by pressing the corresponding lever to receive reward, in this example that is the right side but there were equal left and right trials. On 20% of trials (STOPs; red), the opposite cue light illuminated after the first GO cue that instructed rats to cancel their initial response in the direction of the first cue and instead respond in the direction of the second cue. Left and right trials were randomized. There were 4 basic trial-types: GO-left; GO-right; STOP-Right-go-Left; STOP-Left-go-Right. **b**, **c** Viral expression and recording sites (Paxinos and Watson, 2006). Percent correct (**d**) and reaction times for correct and error trials (**e**) on GO and STOP trials averaged over all recording sessions. **f** Conflict Score (difference between STOP and GO trials for reaction time (STOP-GO/ STOP + GO) and accuracy (GO − STOP/GO + STOP) averaged together as a composite measure of how well control and HDAC5 groups resolved conflict. Open circles are individual rats (Control = 5; HDAC5 = 9). Error bars represent ± SEM. Asterisks represent planned comparisons revealing statistically significant differences between groups (Tukey posthoc *p* < 0.05). Reaction times over sessions for control (**g**) and HDAC5 (**h)** rats.

Electrodes were manufactured and implanted as in prior experiments [46–50]. Rats (control: n = 10, HDAC5 overexpressed: n = 9) received bilateral injections (0.75 μl/hemisphere) of either AAV2-mHDAC5 (experimental) or AAV2-GFP (control) in their insula [anteroposterior (AP): +1.5 mm; mediolateral (ML): ±5.0 mm; dorsoventral (DV): -5.0 mm]. Neural recordings were obtained from 5 control rats [1 female (right hemisphere) and 4 males (2 left and 2 right hemispheres] and 9 HDAC rats [3 females (2 left and 1 right hemispheres) and 6 males (3 left and 3 right hemispheres)]. Five animals initially trained on the tasks did not contribute to neural recordings due to health issues or equipment malfunction. Although we recorded from both hemispheres and sexes, our sample was not large enough to determine sex- or hemisphere- difference. Hemispheric specialization is not commonly studied in rats and sex differences have not been examined during performance of a rat STOP-signal task. In the context of STOP-signal performance in humans, most studies report no behavioral differences between sexes [51–54] and in other types of studies in non-rodent species, lateralized effects in insula have been observed in some cases [55–57] but not others [58]. In vivo confirmation of HDAC5 overexpression was previously illustrated by Li et al. [10], and was also illustrated in our experimental animals (Fig. 1c). Virus delivery occurred at a rate of 0.15 μl/min via Hamilton syringes. Injection needles were left in place for one minute. Following injection, rats were implanted with a drivable bundle of eight 25 µm diameter FeNiCr wires (Stablohm 675, California Fine Wire), counterbalanced across hemispheres (AP: +1.5 mm; ML: ±5.0 mm DV: −5.0 mm) [59, 60].

Rats were anesthetized with isoflurane and transcardially perfused using 300 mL of 4% paraformaldehyde and were transferred into 30% sucrose PBS solution. After slicing (40 μm), sections were washed for 10 minutes in PBS and then incubated for 1 h in blocking buffer (2% BSA in PBS with 0.3% Triton-x100). The sections were incubated with a primary antibody against HDAC5 (sc-133106, 1:500, Santa-Cruz, TX, RRID: AB_2116793). After washing the sections 3 times in PBS (5 min each), they were incubated with the secondary antibody Alexa 594-labeled anti-mouse (R37121, 1:200, Thermo Fisher Scientific, MD, RRID: AB_2556549) in blocking buffer for 1 h. Sections were washed in PBS and mounted (Fisherbrand™ Superfrost™ Slides, Cat #12-550-15) and cover-slipped with Fluoromount-G (Electron Microscopy Sciences).

Electrode tracks were drawn on coordinate matched printouts and verified and cross-referenced with sheets demarcating electrode advancement and initial placement. The section displayed in Fig. 1b, c is ~1.1–1.2 mm anterior to bregma, and all rats showed evidence of the electrode passing through the 1.1–1.2 mm anterior to bregma plane plus or minus 0.1 mm.

### Stop-change task

All rats were randomly assigned to groups and trained before surgery. During performance of the STOP-change task (Fig. 1a), each trial began with the illumination of the house lights, signaling the rat to perform a nose poke into the central port. This poke triggered a 200 ms pre-cue delay after which a directional light was illuminated on either the rat’s left or right side, remaining on until the rat made a response. In 80% of trials (GO trials), the directional light indicated which direction the rat should respond in by pressing the corresponding lever. In the remaining 20% of trials (STOP trials), a light opposite to the initially cued direction illuminated after a stop-signal delay (SSD, 350–1000 ms) and stayed on until a response was made. During STOP trials, rats were required to inhibit the movement prompted by the first light and instead respond in the direction indicated by the second light. These trials were randomly interspersed with GO trials. The SSD was adjusted on a trial-by-trial basis, starting at 450 ms and increasing or decreasing by 40 ms depending on whether the previous trial was correct or incorrect. After each response, rats had to remain in the fluid well for 200 ms before receiving a reward (10% sucrose). Error trials or premature exits resulted in extinction of house lights and a 4 s inter-trial interval. Overall, there were four trial-types: GO-left, GO-right, STOP-left-go-right and STOP-right-go-left.

During shaping, rats were trained to poke centrally before pressing either lever for reward (2–4 sessions). Rats were then introduced to GO trials in which a cue light on either side indicated which lever the rat needed to press to receive reward (~25 sessions). Once criterion was reached for Go trials (80% correct), STOP trials were introduced two per day until the proportion reached 20%. Surgery was performed once average percent correct was at least 80%. Rats were allowed two weeks to recover after surgery and the data shown in the paper are from recording sessions (2-3 months) to better relate neural firing to behavior. There were no differences between groups in terms of trials performed [t(12) = 0.59, p = 0.57] or accuracy [GO: t(12) = 0.71, p = 0.49); STOP: t(12) = 0.32, p = 0.76)] on the last day of training before surgery.

### Single-unit recordings

Electrodes were advanced 40 µm daily. Neural signals (OmniPlex; Plexon) from electrode wires were amplified 20x by an op-amp headstage located on the electrode array. Immediately outside the training chamber, wideband signals were passed through a digital headstage (Digital Headstage Processor; Plexon) where they were digitized at 40 kHz, and bandpass filtered in control software (PlexControl; 250–8000 Hz) to isolate spike activity.

### Data analysis

Units were sorted via Offline Sorter software (Plexon) using template matching and analyzed in Neuroexplorer (Plexon) and MATLAB (R2020b; MathWorks). The first two principal components were used to identify action potential waveforms. Template matching was performed based on average waveform shape. Tolerance was adjusted so that invalid waveforms were not included. Activity was examined during the period between the initial cue light illumination and lever press (response epoch) and the 2000 ms period prior to cue light illumination (baseline epoch). Behavioral data was analyzed using ANOVA session averages to better correspond behavior to neural activiy. Factors included group (control vs. HDAC5), trial-type (GO vs. STOP) and session. Reaction time analysis had an additional factor of correctness. A priori planned comparisons were used to determine differences between performance and reaction times.

## Results

### HDAC5 overexpression disrupts accuracy and makes rats faster on GO trials

**Fig. 2:**
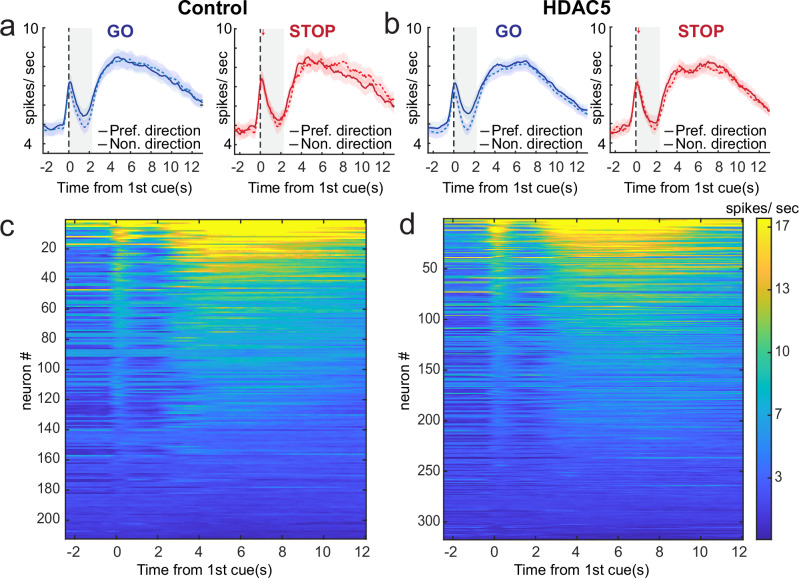
Insula neurons fire during cues and rewards. **a**, **b** Population histograms for all controls (n = 211) and HDAC5 (n = 317) cells aligned to first cue onset for correct GO (blue) and STOP (red) trials. Line thickness indicates preferred direction (thick/solid = preferred; thin/dashed = nonpreferred), which was defined as the direction that elicited the strongest response across correct GO trials during the response epoch (first cue light onset to lever press) for each neuron. Ribbons represent SEM. **c**, **d** Heatmap of average neuronal activity across all rewarded trials, aligned to first directional cue light onset and sorted by average firing over the trial for all control (n = 211) and HDAC5 (n = 317) cells. Each line represents a cell.

Rats were more accurate on GO compared to STOP trials, (Fig. 1d; main effect of trial-type: F(1,314) = 144.7, p < 0.0001), however across sessions, HDAC5 rats were less accurate overall (Fig. 1d; main effect of group: F(1, 314) = 13.6, p = 0.0003), leaning toward a stronger disruption on STOP trials (Fig. 2a; group x trial-type: F(1, 314) = 2.4, p = 0.12). Planned comparisons to test the hypothesis that HDAC5 altered performance on STOP trials showed significant differences between control and HDAC5 rats on STOP trials (t(198) = 3.3, p = 0.001), but not GO trials (t(198) = 1.9, p = 0.07). There were no main (F(21, 314) = 1.4, p = 0.15) or interaction effects (trial-type x session: F(21, 314) = 1.0, p = 0.45) with session, and no interaction with treatment (treatment x session F(20, 314) = 0.61, p = 0.91; treatment x trial-type x session F(20, 314) = 0.58, p = 0.93).

Over both groups, reaction times were faster on GO compared to STOP trials (Fig. 1e; main effect of trial-type: F(1,545) = 54.3, p < 0.0001) and there was no main effect of treatment (F(1, 545) = 1.5, p = 0.22), however, there was a significant interaction between treatment x trial-type x session (F(20, 545) = 1.7, p = 0.02) and treatment x accuracy x session (F(20, 545) = 2.0, p = 0.005). Planned comparisons show that HDAC5 rats were significantly faster on GO correct trials only (Fig. 2b; t(198) = 2.1, p = 0.04). To explore the significant interactions with session we plotted reaction times averaged across GO and STOP error trials and STOP correct trials against session number (Fig. 1g, h). Both groups became significantly more reactive to the first cue (Control: r^2^ = 0.19, p < 0.001; HDAC5: r^2^ = 0.10, p < 0.001) over sessions, but only controls also became significantly faster on STOP trials (Control: r^2^ = 0.06, p < 0.05; HDAC5: r^2^ = 0.005, p = 0.04). We interpret these results as a reflection of HDAC5 altering the ability to resolve conflict as reactivity to the first cue increased over sessions.

These data suggest that rats in both groups could resolve conflict between competing movements signaled by the first and second cue light by stopping and redirecting behavior on STOP trials. However, HDAC5 rats were more strongly driven by the first cue and less likely to inhibit behavior in response to the second cue. In a final behavioral analysis, we computed a composite score by computing the difference between STOP and GO trials for reaction time (STOP-GO/ STOP + GO) and accuracy (GO-STOP/GO + STOP), and averaging them together, to provide a single measure reflecting how well conflict was resolved (higher scores indicate worse cognitive control). We found that HDAC5 conflict scores were significantly higher compared to controls (Fig. 1f; treatment: F(1, 157) = 6.5, p = 0.01; session: F(21, 157) = 6.5, p = 0.01; treatment x session: F(20, 157) = 0.8, p = 0.73).

### GO and STOP directional selectivity was stronger and weaker in HDAC5 rats, respectively

We recorded from 211 and 317 cells from control (male: n = 7, n = 47, n = 21, n = 71; female: n = 65) and HDAC5 (male: n = 49, n = 27, n = 49, n = 42, n = 10, n = 46; female: n = 13, n = 33, n = 40) groups during the STOP-change task. As previously reported, we observed increases in firing to cues and the anticipation of reward, and that firing during the response epoch (i.e. first cue to lever press) reflected the direction of the behavioral response [59]. Increasing (i.e., excited) and decreasing (i.e., inhibited) populations of cells were observed, with more cells significantly (Wilxoxon; p < 0.05) increasing firing compare to those that significantly decreased firing (Control: Increasing = 44, Decreasing = 17, chi-square = 11.9, p = 0.0005; HDAC5: Increasing = 115, Decreasing= 56, chi-square = 20.3, p < 0.0001) relative to baseline. The proportion of increasing to decreasing cells did not differ between the groups (Chi-square = 0.30, p = 0.59).

Average firing rate over trial time is illustrated for each individual neuron (i.e., heat plot; Fig. 2c, d) and averaged across the entire population in Fig. 2a, b. The population histograms (Fig. 2a, b) are broken down by trial-type and cell’s preferred direction, where “preferred direction,” or “movement in the cell’s response field,” is defined by the direction of movement (left or right) that produced higher firing during the response epoch on GO trials because they unambiguously signaled response direction [59, 61].

**Fig. 3:**
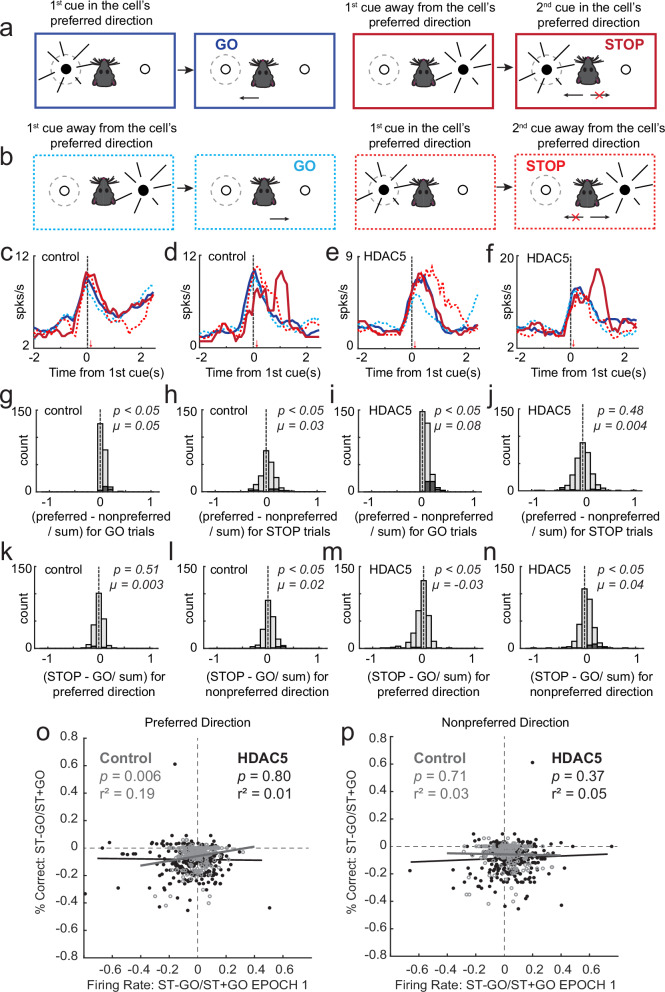
GO and STOP directional selectivity was stronger and weaker in HDAC5 rats. llustration of trial-types for response made into (**a**) and away from **b** the neuron’s preferred direction. Gray dashed circle denotes response field. Four single cell examples from control (**c**, **d**) and HDAC5 (**e**, **f)** rats. Blue = GO; Red = STOP; Thick-Solid = Preferred Direction; Thin-Dashed = Nonpreferred Direction. Distribution of directional indices (preferred – nonpreferred/ preferred + nonpreferred) computed during the response epoch for correct GO (**g**, **i**) and STOP (**h**, **j**) trials (Wilcoxon test, µ = mean) for control (**g**, **h**; n = 211) and HDAC5 (**i**, **j**; n = 317) cells. **k**, **n** Distribution of STOP indices (STOP-GO/ STOP + GO) computed during the response epoch for correct preferred (**k**, **m**) and STOP (**l**, **m**) trials (Wilcoxon test, µ = mean) for control (**k**, **l**; n = 211) and HDAC5 (**m**, **n**; n = 317) cells. Dark gray bars in distributions indicated significant within-cell differences (Wilcoxon; p < 0.05). Correlation between firing rate and percent correct STOP indices for Control and HDAC5 for the preferred (**o**) and nonpreferred (**p**) directions.

As reported previously, the neural manifestation of conflict observed in the behavior can be realized by looking at the strength of directional signals during STOP trials (Fig. 3a, b; red thick vs thin red). That is, competition between the two directions resulted in weaker directional signals on STOP trials when examining average firing over all neurons [48–50, 61, 62]. This is a product of the activity of neurons not accurately reflecting the correct movement because firing has not adjusted to reflect the inhibition and redirection of behavior. This reflects that the majority of neurons either did not signal either direction (i.e., poor encoding of direction; e.g., Fig. 3c) or continued to signal the first cue (i.e., GO direction perpetuated the wrong direction; e.g., Fig. 3e), as opposed to accurately encoding the direction signaled by the STOP cue (e.g., Fig. 3d, f).

To quantitatively assess the strength of directional signals during performance of both STOP and GO trials, we computed a directional selectivity index for each neuron by subtracting average firing rate during the response epoch for movements made into and away from the preferred direction (preferred − nonpreferred/preferred + nonpreferred). We found that the distribution of directional indices during STOP trials was significantly shifted in the positive direction in control (Wilcoxon signed-rank test, µ = 0.03, p < 0.05; Fig. 3h), but not HDAC5 rats (Wilcoxon signed-rank test, µ = 0.004, p = 0.48; Fig. 3j), and that the two distributions were significantly different from each other (Wilcoxon rank-sum test, z-value = 2.11, p = 0.03) suggesting that directional signals after HDAC5 were not resolved in insula by completion of the behavioral response. Notably, if we examine selectivity over 100 ms bins (Wilcoxon), we see that the difference in HDAC5 rats becomes significant at 2.55 seconds, whereas, in controls, it becomes significant 1 second earlier. Importantly, two and a half seconds is roughly the reaction time of the animals. Thus, not only is the signal weaker, but it is delayed to the extent that it is unlikely to contribute to the generation of the movement.

We also observed that GO directional distributions were significantly more strongly shifted after HDAC5 overexpression (Wilcoxon rank-sum test, z-value = 3.90, p < 0.0001; Fig. 3g versus I), thus in addition to firing in insula not accurately reflecting the correct direction on STOP trials, direction was more strongly encoded during GO trials. Theoretically, increased and decreased directional signaling in insula could produce faster GO reaction times and worse performance on STOP trials as observed in HDAC5 rats.

To quantitatively assess the difference between STOP and GO firing, we computed a STOP index (STOP − GO/ STOP + GO) for movements made into and away from the preferred direction. We found that the distribution of STOP indices was significantly shifted in the positive direction in both control (Wilcoxon signed-rank test, µ = 0.02, p < 0.001; Fig. 3l) and HDAC5 rats (Wilcoxon signed-rank test, µ = 0.04. p < 0.001; Fig. 3n) in the nonpreferred direction, and that the two distributions were significantly different from each other (Wilcoxon rank-sum test, z-value = 2.35, p = 0.02). Interestingly, in the preferred direction, distributions in HDAC5 rats (Wilcoxon signed-rank test, µ = −0.03, p = 0.001; Fig. 3m), not controls (Wilcoxon signed-rank test, µ = 0.003, p = 0.51; Fig. 3k), was significantly negatively shifted indicating that the majority of HDAC5 neurons fired less on STOP trials compared to GO trials, however control and HDAC5 distributions were not significantly different from each other (Wilcoxon rank-sum test, z-value = 1.56, p = 0.12). Together, these results are consistent with the idea that directional signals in HDAC rats are more conflicted, potentially leading to worse behavior.

To determine the relationship between firing and behavior on STOP vs. GO trials we plotted the STOP index for firing against the STOP index for percent correct (Fig. 3o) and reaction time (Fig. 3p). The STOP index is a normalized reflection of how well rats were able to inhibit behavior during each recording session. We found that in the preferred direction there was a significant positive correlation between firing and accuracy in controls only (r^2^ = 0.19; p < 0.05). This relationship did not exist when examining reaction times in that quicker resolution of behavior on correct trials was not correlated with stronger differences between STOP and GO firing (control preferred direction: r^2^ = 0.5, p = 0.47; control nonpreferred direction: r^2^ = 0.06, p = 0.39; HDAC5 preferred direction: r^2^ = −0.01, p = 0.80; HDAC5 nonpreferred direction: r^2^ = 0.3, p = 0.61). We conclude that fluctuations in how well animals resolve conflict from session to session is correlated with how differential the firing is on STOP and GO trials, suggesting that insula normally contributes to the balance between inhibition and drive.

### Neural modulation by reward history was not impacted by HDAC5 manipulation

**Fig. 4:**
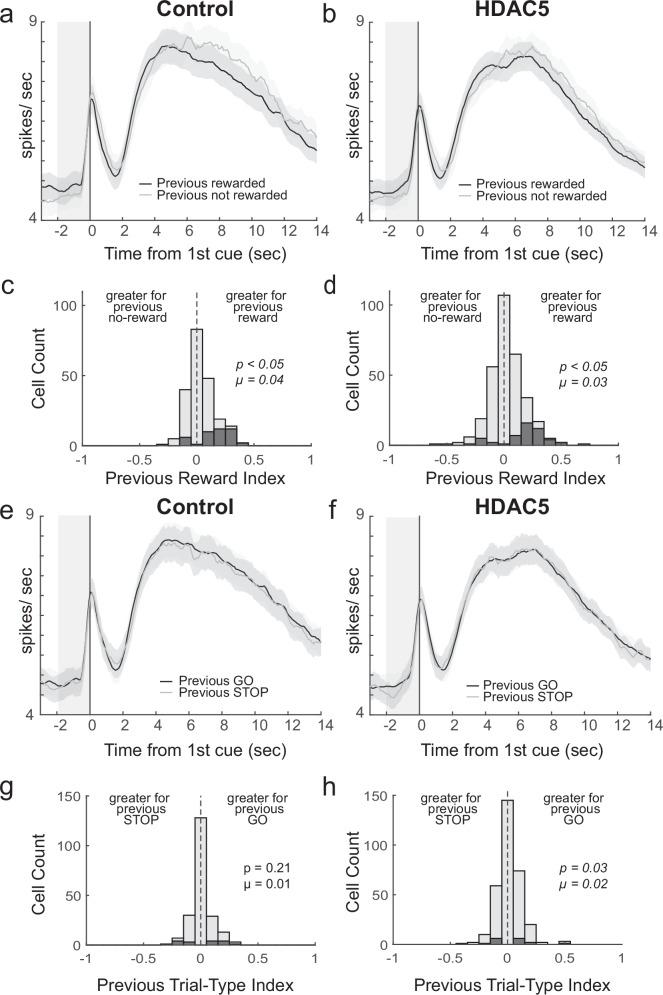
Neural modulation by trial history was not impacted by HDAC5 manipulation. Average firing over direction for correct trials aligned to first cue onset split by trials that followed a rewarded trials (i.e., correct) or not rewarded trials (i.e., chose wrong direction and was not rewarded) for control (**a**; n = 211) and HDAC5 (**b**; n = 317) cells. Note that the previous trial could have been a GO or a STOP and current trial direction was collapsed to increase the sample. Black = previous trial rewarded; Gray = previous trial not rewarded. **c**, **d** Distribution of Previous Reward indices (previous trial rewarded – previous trial not rewarded/previous trial rewarded + previous trial not rewarded) for control and HDAC5 computed during the pre-cue epoch (2 s before first cue) across all correct trials (p = Wilcoxon test, µ = mean). **e**, **f** Average firing over direction for correct trials aligned to first cue onset split by trials that followed correct GO or STOP trials for control (n = 211) and HDAC5 (n = 317) cells. Black = previous GO trial; Gray = previous STOP trial. **g**, **h** Distribution of Previous Trial-Type indices (previous GO – previous STOP/sum for control and HDAC5 computed during the pre-cue epoch across all correct trials (p = Wilcoxon test, µ = mean). Ribbons represent SEM.

Previously, we have shown that activity preceding the onset of a behavioral trial in anterior insula and NAc are influenced by the identity of the outcome that occurred on the previous trial or a block of trials [60, 61]. Here, we asked if this signal in the insula may serve a similar function during performance of the STOP-change task. To answer this question, we plotted the average firing over all neurons aligned to presentation of the first cue light, separated by whether the response on the preceding trial was rewarded or not. Due to small sample size (i.e., rats performed few errors) and that rats were unaware of the identity of the current trial until it happened, we collapsed across trial-type and response direction. As previously described, firing preceding the start of the trials was higher after rewarded trials for both groups (Fig. 4a, b).

To quantify this, we computed a “previous reward index” for each neuron by subtracting average firing during the baseline epoch when the preceding trial was not rewarded compared to when the preceding trial was rewarded and dividing by the sum (reward − no reward/reward + no reward). We found a significant positive shift for both control (Wilcoxon signed-rank test, µ = 0.04, p < 0.05; Fig. 4c) and HDAC5 (Wilcoxon signed-rank test, µ = 0.03, p < 0.05; Fig. 4d), and no significant difference between them (Wilcoxon rank-sum; p = 0.51).

We also computed a “previous trial-type index” for each neuron by subtracting average firing during the baseline epoch when the preceding trial was a correct GO compared to when the preceding trial was a correct STOP trial (GO − STOP/ GO + STOP). We found a significant positive shift for HDAC5 only (Fig. 4e–h; Wilcoxon signed-rank test, HDAC5: µ = 0.02, p = 0.03; Control, µ = 0.01, p = 0.21), but there was no significant difference between the two distributions (Wilcoxon rank-sum; z-value = 0.46, p = 0.64).

## Discussion

Insula has been traditionally described as an interoceptive center [63–65], has strong links to addiction [66–77], and contributes to reward-related functions and decision-making [78–91]. Previous reports in humans demonstrate stronger activation on STOP or NOGO trials compared to GO trials or failures [92], consistent with its proposed role in the salience network [93]. Higher bold signals on STOP trials might reflect some sort of response inhibition or conflict signal but might also reflect that single neurons encoding opposite and competing responses (i.e., conflict) are simultaneously activated. Our data shows that average higher firing across directional responses on STOPs partially reflects that directional signals were slow to resolve resulting in near simultaneous activation of both prepotent and redirected behaviors. Whether this signal is a reflection of increased salience or attention, or perhaps other interoceptive signals, is still unclear, though recent work has suggested that anterior insula contributes to impulse control via mechanisms related to arousal, behavioral adjustments and motor planning [94, 95].

Importantly, our results also show that conflicted directional signals on STOP trials were adequately adjusted or resolved by the completion of the behavioral response suggesting that insula can contribute to resolution of directional signals on STOP trials in control animals. These observed activity patterns during performance of the STOP-change task are consistent with what we described previously while recording from the same region in insula in rats performing a reward-guided decision-making task that addresses another form of impulsivity known as delay-discounting (i.e., long delays to reward discount its value) [60]. In that study we too found that activity increased to both cues and rewards, and that firing during the response was directional, suggesting a role of the insula in encoding subjective reward preference that may drive impulsivity in terms of delay-discounting [60]. In a subsequent study, we showed that lesions of the same region made rats less stimulus-driven [59]. Overall, these results are consistent with the idea that increased drive on GO trials in HDAC5 rats may contribute to the inability to inhibit behavior on STOP trials.

Also consistent with our previous recording study, activity preceding the onset of the trial was influenced by the outcome of the previous trial [60]. Notably, we have observed very similar signals in NAc in rats performing the identical STOP-change task. Future work is necessary to determine if they are reliant on input from insula [96]. While this signal might be an important function of insula and its downstream firing, it does not appear to be regulated by HDAC5, though other HDACs may play a role [97, 98].

Our work contributes to the literature suggesting that the insula may act as a “catch-all,” filtering inputs related to bodily states, emotions, senses, and past events to project motivationally relevant information to motor planning regions of the brain [95, 99–103]. This prioritizing of information may be useful when the cognitive load is high or uncertain, supporting action plans and inhibiting those that will lead the organism away from overall goals [104]. Interestingly, human studies have shown a strong positive correlation between self-control and interoception, perhaps inviting a new way for researchers to conceptualize impulse related disorders [105, 106].

In conclusion, we show that HDAC5 overexpression strengthens and weakens GO and STOP control signals, respectively, tipping the balance away from self-control and towards prepotent, automatic behavior, resulting in increased motor impulsivity, which is a hallmark symptom of aging, addiction and psychiatric illness. Future work should explore therapeutically targeting HDAC5 in insula to improve executive control, as well as other cognitive, social or emotional factors that might be factored into models of impulsivity [92].

## Data Availability

The datasets generated during the current study are available from the corresponding author on request.
